# Detecting associations between behavioral addictions and dopamine agonists in the Food & Drug Administration’s Adverse Event database

**DOI:** 10.1556/JBA.3.2014.1.3

**Published:** 2014-03-24

**Authors:** Katherine E. Gendreau, Marc N. Potenza

**Affiliations:** Yale University School of Medicine, New Haven, CT, USA

**Keywords:** behavioral addictions, impulse control disorders, Parkinson’s disease, dopamine agonists, pharmaco-vigilance, FAERS

## Abstract

*Background/Aims:* Studies have reported higher prevalences of four behavioral addictions (binge eating, compulsive shopping, hypersexuality, and pathological gambling) in dopamine agonist-treated Parkinson’s disease relative to non-dopamine agonist-treated Parkinson’s. However, recent case-control and epidemiological studies suggest that prevalences of behavioral addictions in dopamine agonist-treated Parkinson’s may be similar to background population rates. This study tests that hypothesis by examining the FDA Adverse Event Reporting System (FAERS) for evidence of these associations, taking into account the potential impact of publicity on reporting rates. *Methods:* FAERS reports in 2004 (pre-publicity for all but pathological gambling) and 2007 (post-publicity for all four behaviors) were analyzed. A threshold consisting of ≥3 cases, proportional reporting ratio ≥2, and *χ*^2^ with Yates’ correction ≥4 was used to detect signals (drug-associated adverse reactions) involving any of five dopamine agonists and any of four behavioral addictions. *Results:* No reports containing compulsive shopping and no signal for binge eating and dopamine agonists were found in either year. A weak signal was found for hypersexuality in 2004, with a stronger signal in 2007. A robust signal was found for pathological gambling in 2004, with a more robust signal in 2007. *Discussion/Conclusions:* These results suggest that publicity may increase reporting rates in the FAERS. Findings for binge eating, compulsive shopping, and hypersexuality suggest that prevalences of these behaviors among those treated with dopamine agonists may be similar to background population rates and thus may not reflect an adverse safety signal. Further investigation of the relationship between dopamine agonists and behavioral addictions is warranted.

## Introduction

Since the 1960s, the US Food and Drug Administration (FDA) has maintained a database, now called the FDA Adverse Event Reporting System (FAERS), containing reported adverse drug reactions (ADRs). As part of the FDA’s post-market drug safety surveillance program, the database is monitored for as yet unsuspected drug/ADR associations. The myriad strengths of spontaneous reporting systems like the FAERS have led to their becoming the mainstay of pharmacovigilance programs, but their limitations are also well recognized (Härmark & Van Grootheest, 2008; Stephenson & Hauben, 2007). Strengths include cost-effectiveness, as well as database structures intended to “cast a wide net”. For example, those submitting reports to the FAERS are encouraged to report all drugs they were taking at the time of the ADR, as well as all phenomena that could have been ADRs. Also, while it is possible to designate any single drug in an FAERS report as the “primary suspect”, there is no way to designate any single ADR as the catalyst for the report. Moreover, in the interest of limiting barriers to reporting, a report can be submitted by any individual or entity – healthcare providers, patients, caregivers, legal professionals, pharmaceutical companies, etc. However, this feature also gives rise to one of FAERS’ limitations – since anyone can submit a report, there are no formal criteria qualifying the information submitted, so accuracy, quality, and completeness vary (Stephenson & Hauben, 2007). The FAERS is also limited in that it measures only the frequency of reporting (i.e., it cannot be used to estimate incidence or prevalence of ADRs), and outside factors such as “dear healthcare provider” letters and publicity have been found to influence reporting rates (Bate & Evans, 2009; Moore et al., 2003).

The first English-language report to suggest a link between the dopamine agonist (DA) pramipexole [used to treat Parkinson’s disease (PD)] and an impulse control disorder [in this case, pathological gambling (PG) – a condition now classified as a non-substance or behavioral addiction] was published in 2003 and received considerable publicity (Driver-Dunckley, Samanta & Stacy, 2003). The authors performed a retrospective database review for PG among 1,884 PD patients seen in a 12-month period at a PD research center. They identified nine subjects, eight of whom were taking pramipexole at the time of PG onset. The ninth subject was taking pergolide, another DA. The authors did not specify by which criteria PG diagnosis was made and did not evaluate statistical significance.

Prior reports suggesting links between dopaminergic treatment for PD and PG differed from the 2003 report in that they did not receive publicity and either described hedonistic homeostatic dysregulation, a neuropsychological behavioral condition associated with substance (primarily levodopa) misuse and/or addiction (Giovannoni, O’Sullivan, Turner, Manson & Lees, 2000; Gschwandtner, Aston, Renaud & Fuhr, 2001; Serrano-Dueñas, 2002); or the dopaminergic drug in question was levodopa (Cools, Barker, Sahakian & Robbins, 2003; Molina et al., 2000).

In 2006, Szarfman, Doraiswamy, Tonning and Levine reported results of their examination of FAERS data from its inception through March 2005 for evidence of the association reported by Driver-Dunckley et al. (2003). Szarfman et al. (2006) found that 39 of 67 reports containing gambling also contained the DA pramipexole. However, the authors did not evaluate the potential effect of publicity resulting from the 2003 report on the reporting rate.

Four studies linking PG and three additional behavioral addictions to DA treatment were published and received considerable publicity in 2005 and 2006. Two of these studies reported associations between pramipexole and PG, as well as binge eating (BE), compulsive shopping (CS), and hypersexuality (HS) (Dodd et al., 2005; Pontone, Williams, Bassett & Marsh, 2006). The remaining two studies reported associations between DAs in general (not just pramipexole) and CS, HS, and PG (Voon et al., 2006; Weintraub et al., 2006).

There are several factors to consider in the evaluation of the reported associations. First, Dodd et al. (2005) described a case series. Second, while the authors of the three 2006 studies [i.e., Pontone et al. (2006), Voon et al. (2006), Weintraub et al. (2006)], as well as a large, multi-center, international study in 2010 (Weintraub et al., 2010) conducted cross-sectional trials in North America, utilized validated screens and/or DSM-IV diagnostic criteria and provided statistical analyses, none of them compared prevalences of BE, CS or HS among either DA-treated or non-DA-treated PD patients to prevalences in the general population. While prevalences found among selected cohorts are not directly comparable to prevalences found in the general population, it is interesting to note that when current prevalences (time frames ranged from active to six months) of BE, CS, and HS among non-DA-treated PD patients found in these four studies are compared to point prevalences reported in the general population, the former appear to be lower than the latter [1.7% vs. 6.6% (BE); from 0% to 2.9% vs. 5.8% (CS); and from 0% to 1.7% vs. 5% (HS)], although prevalence estimates for some of these conditions/behaviors (e.g., HS) have not been well examined in large community samples. That being said, point prevalences of BE, CS and HS among DA-treated PD patients reported in these four studies appear to be similar to those reported in the general population [5.6% vs. 6.6% (BE); from 0.7% to 7.2% vs. 5.8% (CS); and from 4.4% to 6.3% vs. 5% (HS)] (Black, Kehrberg, Flumerfelt & Schlosser, 1997; Grucza, Przybeck & Cloninger, 2007; Koran, Faber, Aboujaoude, Large & Serpe 2006; Shaffer, Hall & Vander Bilt, 1999). Additionally, none of these four cross-sectional studies excluded those who had experienced these conditions prior to PD onset. A history of behavioral addictions prior to disease onset has been found to be associated with the presence of a behavioral addiction post-disease onset (Evans, Strafella, Weintraub & Stacy, 2009), and Weintraub et al. (2006) found that 36.4% of the active cases identified in their study had experienced the same behavioral addiction prior to PD onset.

These observations suggest the possibility of alternate interpretations of the apparent elevation in the prevalences of BE, CS, and HS in DA-treated PD patients. For example, it is possible that the dopamine depletion characteristic of PD could be accompanied by reduced frequencies of certain behaviors in which dopamine plays a role, and DA therapy brings the prevalences of these behaviors back to background rates. If this were the case, then while there could be evidence of such associations in the FAERS post-media coverage due to publicity-stimulated reporting, there might be no evidence in the FAERS pre-media coverage. Given that FAERS data dating back to 2004, i.e., before the associations between DAs and BE, CS, and HS (but not PG) were reported and publicized, are publicly available on the FDA’s website, these data provide an opportunity to test that hypothesis. For this analysis, FAERS data were examined at two time points, 2004 and 2007, for evidence of associations between DAs and BE, CS, HS, and PG. Given the observations listed in the previous paragraph, we hypothesized that there would be evidence of these associations in 2007, post-publicity of potential adverse effects, and that, with the exception of PG, such associations would be weaker or not evident in 2004.

## Methods

### Participants

The ADR and drug data used in this analysis were obtained from the FDA’s FAERS database. FAERS data from January 1, 2004 through the most recently completed quarter are available on the FDA’s website.

### Procedure

Each report to the FAERS is reviewed and ADRs are recoded for data entry using the standardized, or “preferred”, terms found in the Medical Dictionary for Regulatory Affairs (MedDRA). We did not find the preferred term compulsive shopping or any relevant preferred terms (shop-lifting was excluded) containing the text strings buy, shop, or spend, so only three relevant preferred terms were selected for this analysis: binge eating, hypersexuality, and pathological gambling. For each of those three terms, a group of clinically related preferred terms was also selected (see Table 1). FAERS reports listing one of the following five orally administered DAs – bromocriptine, cabergoline, pergolide, pramipexole, and ropinirole – as well as one or more of these preferred terms, were analyzed. The drugs were captured by using searches for exact spelling text strings for trade and generic names as well as for a variety of close misspellings. All initial reports in 2004 (i.e., pre-publicity for BE, CS, and HS, but post-publicity for PG) and 2007 (i.e., post-publicity for all four conditions/behaviors) were considered regardless of categorization of DA as primary suspect. A one-year time-period was selected in each case as there is only one year of FAERS data available prior to 2005 and a comparable time-period was selected for comparison status post the 2005 and 2006 publications mentioned above. Given that there is no limit to the number of follow-up reports allowed per initial report, nor to the frequency of reporting the same ADR for the same person, follow-up reports were excluded.

### Measures and statistical analysis

The data analysis algorithm chosen was the proportional reporting ratio (PRR). The PRR compares the frequency with which a particular ADR is reported for individuals taking a specific drug/type of drug to the frequency with which the same ADR is reported for individuals taking any other drug. It is computed as [*a*/(*a* + *c*)]/[*b*/(*b* + *d*)], where *a* is the number of exposed cases, *b* is the number of unexposed cases, *c* is the number of exposed non-cases, and *d* is the number of unexposed non-cases (Evans, Waller & Davis, 2001). It is straightforward to perform and interpret, commonly used, and is arguably more sensitive than other, more complex data-mining algorithms (Hauben, Reich & Gerrits, 2006). For this analysis, the threshold established by Evans et al. (2001) of three or more cases, PRR of at least two, and a *χ*^2^ with Yates’ correction of at least four was considered a signal suggesting a potential hazard warranting further investigation – for example, review of the full reports, which could contain comorbidities or other potentially responsible medications. Confidence intervals and *p*-values were also calculated. All statistical analyses were performed using SAS 9.2 (Cary, NC, USA).

**Table 1.** Preferred terms (ADRs) included in analysis

Primary preferred term	Clinically-related preferred term
Binge eating	Eating disorder
	Eating disorder symptom
	Bulimia
Hypersexuality	Excessive masturbation
	Excessive sexual fantasies
	Exhibitionism
	High-risk sexual behavior
	Libido increased
	Sexual abuse
	Sexual activity increased
	Sexual offense
Pathological gambling	Gambling

ADR = adverse drug reaction.

*Note:* We found no preferred terms in 2004 or 2007 that could be categorized as compulsive shopping.

### Ethics

All authors conformed to the highest standards of ethical conduct in the submission of accurate data, acknowledging the work of others and divulging potential conflicts of interest. As the study involved the use of publicly accessible, de-identified data, signed informed consent was not necessary for this study. Given the use of de-identified data, the study is exempted from IRB review under federal regulation 45 CFR Part 46.101(b).

## Results

Results are summarized in Table 2. In 2004, i.e., pre-publicity for BE, CS, and HS, out of 251 reports containing BE, none also contained a DA (PRR = 0; *χ*^2^ = 0.4). We found no reports containing ADRs that could be categorized as CS. Of 91 reports containing HS, four also contained at least one DA (PRR = 10; *χ*^2^ = 22.6); and of 33 reports containing PG, 28 also contained at least one DA (PRR = 1,201; *χ*^2^ = 4,906.9).

In 2007, i.e., recent post-publicity for all four behavioral addictions, of 328 reports containing BE, six also contained at least one DA (PRR = 2; *χ*^2^ = 2.0). Again, we found no reports containing ADRs that could be categorized as CS. Of 95 reports containing HS, 30 also contained at least one DA (PRR = 50; *χ*^2^ = 943.8); and of 184 reports containing PG, 170 also contained at least one DA (PRR = 1,304; *χ*^2^ = 16,752.8).

## Discussion

This analysis was undertaken based upon the following premises and hypotheses: 1) the FAERS captures unsuspected drug/ADR associations; 2) publicity may increase ADR reporting rates. Based on these notions, detection of a strong signal for DAs and PG in 2004, given that it was publicized in 2003, was hypothesized; detection of an equally large or stronger signal for DAs and PG in 2007 was also hypothesized, given ongoing publicity. The results of this analysis are consistent with these hypotheses. Based on observations discussed above, it was also hypothesized that no or weak signals would be detected for DAs and BE, CS and HS in 2004. The results of this analysis for BE, CS and HS are consistent with this hypothesis, with no signal found for BE or CS, and a weak signal found for HS. Finally, it was hypothesized that, in the setting of increased publicity, signals would be detected for DAs and BE, CS and HS in 2007. However, while the results of this analysis for HS were consistent with this hypothesis, signals worthy of further consideration were not found for BE and CS in 2007.

**Table 2.** Frequencies and signal threshold criteria for behavioral addictions and dopamine agonists in the FDA’s FAERS Database in 2004 and 2007

Adverse drug reaction	2004					2007				
	(*N* = 199,754)					(*N* = 254,162)				
	DA	No DA	PRR	95% CI	*χ*^2^	DA	No DA	PRR	95% CI	_*χ*_2
	(*n* = 927)	(*n* = 198,827)			(Yates’)	(*n* = 2,345)	(*n* = 251,817)			(Yates’)
Binge eating	0	251	0	0.0–4.1	0.4^*^	6	322	2	0.8–4.6	2.0^*^
Compulsive shopping	0	0	–	–	–	0	0	–	–	–
Hypersexuality	4	87	10	3.1–27.8	22.6^**^	30	65	50	31.5–77.7	943.8^**^
Pathological gambling	28	5	1,201	443.5–3,534.8	4,906.9^**^	170	14	1,304	740.6–2,342.1	16,752.8^**^

FDA = Food & Drug Administration, FAERS = FDA Adverse Event Reporting System, DA = dopamine agonist, CI = confidence interval, PRR = proportional reporting ratio.

*Note:* The minimum criteria for a signal are: three or more cases, PRR of two or more, and *χ*^2^ with Yates’ correction of at least four. The crude PRR is computed as [*a*/(*a* + *c*)]/[*b*/(*b* + *d*)], where *a* is the number of exposed cases, *b* is the number of unexposed cases, *c* is the number of exposed non-cases, and *d* is the number of unexposed non-cases.

^*^ Both Fisher’s exact and Yates’ correctedp-values > 0.1.

^**^ Both Fisher’s exact and Yates’ corrected p-values < 0.001.

Taken together with the prevalences found by Pontone et al. (2006), Voon et al. (2006), and Weintraub et al. (2006, 2010), one possible interpretation of these results is twofold. First, PD itself may confer decreased risks for the development of behavioral addictions, and second, DA therapy may bring the risks of developing these behaviors back up to baseline population rates. If this were the case, drug-naïve PD patients could be expected to develop these behaviors at rates lower than those of the general population, and DA-treated PD patients could be expected to develop these behaviors at rates similar to those of the general population.

Consistent with this interpretation, neither de Chazeron et al. (2011) nor Isaias et al. (2008) found a significant difference between frequencies of behavioral addictions in treated PD patients relative to healthy comparison subjects in their cross-sectional, case-control studies; but, apparently inconsistent with this interpretation, neither Antonini et al. (2011) nor Weintraub, Papay & Siderowf (2013) found a significant difference between frequencies of behavioral addictions in newly diagnosed, drug-naïve PD patients relative to healthy comparison subjects in their cross-sectional, case-control studies.

However, dopamine depletion in PD is gradual, and compensatory escalation in both dose and number of adjunctive medications over time is typical. Perhaps the net effect of escalation in dopaminergic therapy coupled with gradual dopamine loss is behavioral addictions prevalences similar to those in the general population. It is possible that the newly diagnosed, drug-naïve patients had not yet experienced sufficient dopamine loss to demonstrate depressed prevalences of behavioral addictions. Several studies have found longer disease duration to be associated with the presence of a behavioral addiction (Auyeung et al., 2011; Lee et al., 2009, 2010; Lim et al., 2011; Tanaka, Wada-Isoe, Nakashita, Yamamoto & Nakashima, 2013; Weintraub et al., 2006). If, in addition to its impact on movement, dopamine depletion is associated with a reduction in behavioral addictions, then a DA-related rise in the frequencies of these behaviors back to baseline population rates would not necessarily reflect an adverse safety signal.

Alternatively, it is possible that the FAERS either does not always capture unsuspected drug/ADR associations or that there can be a delay between appearance of an association in the general population and its appearance in the FAERS. In 2013, White, Tatonetti, Shah, Altman & Horvitz found evidence of an adverse drug/drug interaction among millions of anonymized search data from several search engines before it became evident in the FAERS. In addition, many people may not expect drug side effects to encompass changes in behavior; this could lower – or potentially even eliminate, in the case of CS – the reporting rates for these behavioral addictions in conjunction with DAs. And while the reports containing BE and at least one DA did not reach the signal threshold in either 2004 or 2007, the number of reports did rise in 2007. In addition to these possibilities, other factors may influence the emergence of behavioral addictions in PD. For example, being unmarried and living in the United States versus Canada were each associated with behavioral addictions in PD. As such, factors unrelated to PD or its treatment may influence the emergence of behavioral addictions in PD and thus confound attribution and reporting.

As was described earlier, the FAERS is designed to “cast a wide net”. The current analysis maximized this design by not restricting reports analyzed to those in which DAs were categorized as the primary suspect, and by analyzing all initial reports, including those submitted by non-healthcare professionals. Also, the preferred terms chosen for analysis included not only those that were clearly clinically related, but also those that were less clear (such as “sexual abuse” and “sexual offense”), as well as those that were less specific (such as “eating disorder”).

Further, the quantity portion (i.e., three or more cases) of the threshold selected in the current study was originally proposed in the context of ADRs with much lower background risks than BE, CS, HS, and PG [e.g., .00012% (toxic epidermal necrolysis) or .0006% (agranulocytosis) versus 6.6% (BE), 5.8% (CS), 5% (HS) or 1.1% (PG) (Begaud, Moride, Tubert-Bitter, Chaslerie & Haramburu, 1994; Black et al., 1997; Grucza et al., 2007; Koran et al., 2006; Shaffer et al., 1999)]. The probability of coincidental reports increases as background risk increases (Begaud et al., 1994). This could impact interpretation of the four 2004 reports containing both HS and a DA as a signal worthy of further investigation.

This analysis has several limitations. In addition to the limitations of spontaneous reporting systems addressed above, these databases may be subject to underreporting (it is estimated that as few as 10% of ADRs are reported), and the possibility exists that reported cases may be different from unreported cases (Begaud et al., 1994). Additionally, this analysis did not examine individual reports for potential confounders such as comorbidities or other medications. The threshold selected in the current study (three or more cases, PRR of at least two, and a *χ*^2^ with Yates’ correction of at least four) also warrants consideration. While this threshold has been established and used in prior studies (Evans et al., 2001), the use of another threshold may have yielded different results. Finally, follow-up reports were excluded based on the rationale that if both initial and one or more follow-up reports included a DA and a behavioral addiction, the frequency of behavioral addictions would be artificially inflated. However, to the extent that follow-up reports included DAs/behavioral addictions for initial reports that did not, those frequencies were not captured by this analysis.

## Conclusions

The findings of the current study are largely consistent with the notion that increased publicity is associated with increased reporting of behavioral addictions in PD in the FAERS database. The absence of signals for DAs and BE or CS, and, prior to publicity, only a weak signal for DAs and HS, is consistent with the notion that prevalences of these behavioral addictions among those treated with DAs may be similar to background population rates and thus may not reflect an adverse safety signal. Further investigation of the relationship between DAs and behavioral addictions in PD is warranted in order to provide appropriate information to patients, care providers and policy makers.
